# Environmental Plasticity of the RNA Content of *Staphylococcus aureus* Extracellular Vesicles

**DOI:** 10.3389/fmicb.2021.634226

**Published:** 2021-03-11

**Authors:** Brenda Silva Rosa Da Luz, Aurélie Nicolas, Svetlana Chabelskaya, Vinícius de Rezende Rodovalho, Yves Le Loir, Vasco Ariston de Carvalho Azevedo, Brice Felden, Eric Guédon

**Affiliations:** ^1^INRAE, Institut Agro, STLO, Rennes, France; ^2^Laboratory of Cellular and Molecular Genetics, Institute of Biological Sciences, Federal University of Minas Gerais, Belo Horizonte, Brazil; ^3^BRM [Bacterial Regulatory RNAs and Medicine] UMR_S 1230, University of Rennes, Inserm, Rennes, France

**Keywords:** membrane vesicle, small regulatory RNA, virulence factors, vancomycin, RNA-Seq, extracellular vesicle, RsaC, RNAIII

## Abstract

The roles of bacterial extracellular vesicles (EVs) in cell-to-cell signaling are progressively being unraveled. These membranous spheres released by many living cells carry various macromolecules, some of which influence host-pathogen interactions. Bacterial EVs contain RNA, which may serve in communicating with their infected hosts. *Staphylococcus aureus*, an opportunistic human and animal pathogen, produces EVs whose RNA content is still poorly characterized. Here, we investigated in depth the RNA content of *S. aureus* EVs. A high-throughput RNA sequencing approach identified RNAs in EVs produced by the clinical *S. aureus* strain HG003 under different environmental conditions: early- and late-stationary growth phases, and presence or absence of a sublethal vancomycin concentration. On average, sequences corresponding to 78.0% of the annotated transcripts in HG003 genome were identified in HG003 EVs. However, only ~5% of them were highly covered by reads (≥90% coverage) indicating that a large fraction of EV RNAs, notably mRNAs and sRNAs, were fragmented in EVs. According to growth conditions, from 86 to 273 highly covered RNAs were identified into the EVs. They corresponded to 286 unique RNAs, including 220 mRNAs. They coded for numerous virulence-associated factors (*hld* encoded by the multifunctional sRNA RNAIII, *agrBCD*, *psmβ1*, *sbi*, *spa*, and *isaB*), ribosomal proteins, transcriptional regulators, and metabolic enzymes. Twenty-eight sRNAs were also detected, including *bona fide* RsaC. The presence of 22 RNAs within HG003 EVs was confirmed by reverse transcription quantitative PCR (RT-qPCR) experiments. Several of these 286 RNAs were shown to belong to the same transcriptional units in *S. aureus*. Both nature and abundance of the EV RNAs were dramatically affected depending on the growth phase and the presence of vancomycin, whereas much less variations were found in the pool of cellular RNAs of the parent cells. Moreover, the RNA abundance pattern differed between EVs and EV-producing cells according to the growth conditions. Altogether, our findings show that the environment shapes the RNA cargo of the *S. aureus* EVs. Although the composition of EVs is impacted by the physiological state of the producing cells, our findings suggest a selective packaging of RNAs into EVs, as proposed for EV protein cargo. Our study shedds light to the possible roles of potentially functional RNAs in *S. aureus* EVs, notably in host-pathogen interactions.

## Introduction

The release of extracellular vesicles (EVs) by living cells is a well-established phenomenon required for intercellular communications and *trans*-kingdom interactions (Brown et al., 2015; Toyofuku, 2019). These spherical membranous particles vary from 20 to 300 nm in diameter and contain macromolecules such as nucleic acids, proteins, lipids, and small metabolites. Initially considered to be trash bags to eliminate unwanted material outside of the cells, they are now widely recognized as protective delivery shuttles of bioactive molecules from donor to recipient cells (Brown et al., 2015; Kim et al., 2015; Gill et al., 2019). The functional characterization of bacterial EVs is of interest due to their capacities to affect bacteria-host cell interactions and bacterial pathogenesis (Kaparakis-Liaskos and Ferrero, 2015; Tsatsaronis et al., 2018). Although the formation of outer membrane vesicles (OMVs) in Gram-negative bacteria was early documented in 1966 (Work et al., 1966), the formation of such structures was disregarded in Gram-positive bacteria until recently. The production of EVs by a Gram-positive bacterium, *Staphylococcus aureus*, was demonstrated in 2009 and, ever since, numereous studies confirmed EV release by other Gram-positive bacteria (Lee et al., 2009, 2013a; Rivera et al., 2010; Prados-Rosales et al., 2011; Brown et al., 2014; Olaya-Abril et al., 2014; Kim et al., 2016a; Liu et al., 2018b).

*Staphylococcus aureus* commonly colonizes the skin or nasal tract of vertebrates, without causing disease (Wertheim et al., 2005). However, it is also one of the main opportunistic pathogen in humans, and a frequent cause of multi-drug resistant nosocomial infections (Ziebuhr, 2001). *S. aureus* is responsible for a wide array of diseases, ranging from minor infections in soft tissues to life-threatening diseases, such as sepsis, meningitis, and pneumonia (Salgado-Pabón and Schlievert, 2014; Tong et al., 2015). The type and severity of infections depend on strain-specific virulence factors, mostly expressed from accessory genetic elements (Gill et al., 2011). Secreted and surface-exposed *S. aureus* virulence factors weaken the host immune response, leading to bacterial immune evasion and pathogenesis (Foster, 2005). EVs could be a vehicle for secretion and surface-display of these molecules and, accordingly, recent studies indicate that *S. aureus* EVs carry important bacterial survival and virulence factors, such as β-lactamases, toxins, and proteins involved in adhesion to host cells (Lee et al., 2009; Gurung et al., 2011; Jeon et al., 2016; Askarian et al., 2018; Tartaglia et al., 2018, 2020; Wang et al., 2018).

Biologically active β-lactamase in *S. aureus* EVs can confer a transient resistance against ampicillin to surrounding sensible bacteria (Lee et al., 2013b). Furthermore, the presence of α-hemolysin inside EVs accelerates host cell death (Thay et al., 2013; Hong et al., 2014), and EV-associated exfoliative toxin A (ETA) induces a characteristic toxicity onto human epithelial cells (Jeon et al., 2016). Moreover, *S. aureus*-derived EVs facilitate the induction and exacerbation of skin and pulmonary inflammations (Hong et al., 2011, 2014; Kim et al., 2012; Jun et al., 2017). EVs-associated molecules can be more efficient than cytoplasmic proteins to elicit an immune response and host-cell toxicity (Hong et al., 2014). In response to antibiotics exposures, EVs increase *S. aureus* adhesion and cell aggregation, and contribute to biofilm formation (He et al., 2017). Recent data highlight the importance of EVs in staphylococcal pathogenesis since EVs derived from various human and animal strains of *S. aureus* share a conserved EV proteome (Tartaglia et al., 2020).

The vast majority of functional studies on bacterial EVs, however, challenged their proteome. Regarding the presence of DNAs and RNAs in EVs, most studies have been conducted on Gram-negative bacteria (Perez Vidakovics et al., 2010; Blenkiron et al., 2016; Koeppen et al., 2016; Bitto et al., 2017; Choi et al., 2017; Malabirade et al., 2018; Yu et al., 2018; Han et al., 2019). OMV-associated RNAs can include messenger RNAs (mRNA), transfer RNAs (tRNA), ribosomal RNAs (rRNA), or small regulatory RNAs (sRNA; Biller et al., 2014; Ghosal et al., 2015; Ho et al., 2015; Sjöström et al., 2015; Blenkiron et al., 2016; Koeppen et al., 2016; Choi et al., 2017; Dauros-Singorenko et al., 2018; Liu et al., 2018a; Malabirade et al., 2018; Tsatsaronis et al., 2018; Frantz et al., 2019). EV-associated RNA cargo, notably sRNAs, can influence host-pathogen interactions, cell-to-cell communications, and bacterial pathogenesis (Dauros-Singorenko et al., 2018; Tsatsaronis et al., 2018; Lee, 2019; Ahmadi Badi et al., 2020; Lécrivain and Beckmann, 2020). For instance, OMVs from *Pseudomonas aeruginosa* can transfer an sRNA into the human airway cells, resulting in IL-8 decrease (Koeppen et al., 2016). Likewise, transfection of OMV-associated sRNAs from the periodontal pathogens *Aggregatibacter actinomycetemcomitans*, *Phorphyromonas gingivalis*, and *Trepanema denticola* into human cells reduced host interleukine release (Choi et al., 2017). The presence of RNAs within Gram-positive EVs has been reported for fewer species (Resch et al., 2016; Dauros Singorenko et al., 2017; Frantz et al., 2019; Rodriguez and Kuehn, 2020). Interestingly, Frantz et al. (2019) recently reported that the EV-associated *rli*32 sRNA of *Listeria monocytogenes* can trigger the induction of a type I IFN response in host cells. This finding supports that Gram-positive EVs can also participate to host-pathogen interactions by dedicated vesicular RNAs. Data about RNA cargo in EVs released by *S. aureus* are scarce, with only two recent reports. While the first provided a partial RNA profile of *S. aureus* MSSA476 EVs without functional analyses (Joshi et al., 2021), the second showed that the uncharacterized RNA content of *S. aureus* Newman EVs likely stimulate the potent IFN-β response observed in cultured macrophage cells (Rodriguez and Kuehn, 2020).

As far as we know, our work is the first example that provides a detailed RNA profile associated to EVs from a reference clinical *S. aureus* strain, HG003. The staphylococcal EV RNA cargo was unveiled by high-throughput RNA sequencing from purified EVs after release by cells grown under various environmental conditions. They include early- and late-stationary growth phases, with or without a sublethal concentration of vancomycin, an antibiotic used to treat multidrug-resistant infections and that influences *S. aureus* EV biogenesis and functions (Hsu et al., 2011; He et al., 2017). The RNA cargo from the EVs was analyzed and compared to the RNA content of the HG003 parental cells.

## Materials and Methods

### Bacterial Strain and Growth Conditions

The *S. aureus* strain used in this work was the model strain HG003 (Herbert et al., 2010), a NCTC8325 derivative, isolated in 1960 from a sepsis patient. HG003 contains functional *rsbU* and *tcaR* genes, two global regulators that are missing in the NCTC8325 parent strain. The HG003 genome is well documented (Sassi et al., 2014), and this strain is widely used as a reference to investigate staphylococcal regulation and virulence (Liu et al., 2018a). HG003 strain was pre-inoculated in BHI broth and grown overnight at 37°C under 150 rpm/min agitation, and then inoculated 0.1% in 500 ml of fresh BHI (125 rpm/min, at 37°C) on a 1 L Scott flask. Bacterial cultures were retrieved after 6 h and 12 h for early- and late-stationary phases, respectively, in the presence or absence of a sub-inhibitory concentration (0.5 μg/ml) of vancomycin (Supplementary Figure S1).

### *S. aureus* EVs Isolation and Purification

Cultures were submitted to EVs isolation and purification, as previously described (Tartaglia et al., 2018, 2020). In brief, for each condition 1 L of bacterial cell culture was centrifuged at 6,000 × *g* for 15 min and filtered through 0.22 μm Nalgene top filters (Thermo Scientific). Then, the culture supernatant fraction was concentrated around 100-fold using the Amicon ultrafiltration systems (Millipore) with a 100kDa filter, and ultra-centrifuged for 120 min at 150,000 × *g* to eliminate the soluble proteins. Next, the suspended pellet was applied to a discontinuous sucrose gradient (8–68%) and ultra-centrifuged at 100,000 × *g* for 150 min. Fractions containing EVs were recovered and washed in TBS (150 mM NaCl; 50 mM Tris-Cl, pH 7.5) for final ultra-centrifugation at 150,000 × *g* (120 min). At last, EVs were suspended in cold TBS and kept at −80°C until use.

### EVs Visualization by Electron Microscopy

Negative staining electron microscopy was performed as previously described (Rodovalho et al., 2020) to investigate the shape and integrity of purified EVs. EVs samples were diluted, and solutions containing between 10^10^ and 10^11^ particles per ml were analyzed. For this, samples were applied to glow-discharged copper EM grids (FF200-Cu) for 30 s, followed by excess solution removal with filter paper. The same process was repeated with 2% uranyl acetate, and samples were observed with a Jeol 1400 transmission electron microscope (JEOL Ltd.), operating at 120 kV.

### Determination of EVs Sizes and Concentrations

Nanoparticle Tracking Analysis (NTA) using an sCMOS camera and a Blue488 laser (Nano Sight NS300) was performed to assess EVs size and concentration. For that, samples were diluted into TBS to achieve optimal concentration and submitted to a constant flux generated by a syringe pump (speed 50), at 25°C. Results were retrieved from 5 × 60 s videos recorded with camera level at 15 and threshold at 5, while other parameters were adjusted as necessary.

### RNA Extraction From *S. aureus* HG003 Whole Cells and Its Derived EVs

RNA extraction was carried out as similar as possible for both cell and EV samples. Bacterial RNA extraction was performed from 10 ml culture pellet. The samples were mixed with glass beads in 300 μl lysis buffer (0.5% SDS w/v, 30 mM sodium acetate; 1 mM EDTA) and 400 μl phenol (acid buffered at pH 5.0) at 65°C. Mechanical lysis was accomplished with 2 cycles of 30 s in Precellys at 6,500 rpm. For EV sample RNA extraction, particles isolated from the equivalent of 800 ml bacterial culture were mixed with 300 μl of lysis buffer and 400 μl phenol at 65°C, the same volumes used for cell RNA extraction. Since EVs lack the thick layer of peptidoglycan (PGN) found in the bacterial cell wall, mechanic lysis was not necessary and was achieved with lysis buffer. EV and EV-producing cell samples were incubated for 10 min at 65°C, being homogenized by vortex every minute. Next, samples were centrifuged during 10 min 13,000 rpm, 4°C, and the upper phase was recovered to a new tube. All samples were mixed with additional 400 μl of phenol at 65°C, and the previous steps were repeated. Then, 400 μl of phenol:chloroform 1:1 was added, followed by two times addition of 400 μl pure chloroform, repeating the step of upper phase recovery, mixture and centrifugation (5 min at 13,000 rpm, 4°C). Subsequently, 1.5 volumes of ice-cold 100% ethanol and 10% volume of NaAc were added and the mix was stored at −20°C overnight. Samples were centrifuged at 13,000 rpm for 30 min at 4°C, and the pellets were washed twice with 1 ml of cold 70% ethanol. Finally, the pellets were dried with a SpeedVac concentrator for 2 min and dissolved in RNase-free water. The quality and quantity of the RNAs were verified by Nano Drop, agarose gel, and Bioanalyzer (Agilent). Samples were kept at −80°C until use. No RNase treatment was applied to bacterial cell or to EV samples before RNA extraction.

### RNA Sequencing

The RNA samples were sent to ViroScan3D® (Lyon, France) for DNA removal, ribosomal RNA depletion and RNA sequencing. Total RNA samples were submitted to a DNase treatment with RNase-Free DNase Set (Qiagen) according to manufacturer’s instructions. Then, the samples quantified using the Quantifluor RNA system (Promega), and qualified using RNA Nano Chip on Bioanalyzer 2100 (Agilent) for the EV-producing cell samples, and on the SS RNA system on Fragment Analyzer (AATI) for the EVs samples. RNA samples were then submitted to the standard protocol Ovation Universal Prokaryotic RNA-Seq, Nugen, Anydeplete rRNA, library preparation. RNA quantity used for library preparation are displayed in Supplementary Table S1. The quality of libraries was assessed with the Quantifluor DNA system (Promega) and qualified with the HS-NGS system on Fragment Analyzer (Aati). The insert mean size of the libraries was 0.34 kp for the EV-producing cell samples, and 0.45 kp for the EV samples (Supplementary Table S1). Sequencing was performed with Illumina, NextSeq500, 75 cycles, single-read, High Output. For each experimental condition, three biological replicates were sequenced. EV-producing cell samples ranged from 9 to 27 million pair-end reads per sample, and EV samples ranged from 30 to 67 million pair-end reads per sample. Reads mapping to the reference genome ranged from 8 to 26 million, and from 0.38 to 27 million reads for EV-producing cells and EV samples, respectively. Basic statistics of the RNA-Seq data are displayed in Supplementary Table S1.

### Transcriptome Analysis

The reads were cleaned and trimmed with Trim-Galore (Martin, 2011) using the default parameters. Reads were mapped with Bowtie2 (Langmead and Salzberg, 2013) in local mode against two staphylococcal genomes used as references: the NCTC 8325 (NC_007795.1) reference genome with sRNA annotation from SRD (Sassi et al., 2015) and the HG003 genome (GCA_000736455.1) for the non-annotated genes in NCTC8325 genome. Genes were counted with FeatureCounts (Liao et al., 2014) with the strand, the multi mapping, and the overlapping options.

A list of differentially expressed RNAs was obtained by EdgeR (Robinson et al., 2009) embedded in SARTools (Varet et al., 2016). The threshold of statistical significance was set to 0.05, with the adjustment method of Benjamini-Hochberg. RNA coverage was calculated with Bedtools coverage (Quinlan and Hall, 2010). RNAs with ≥ 90% coverage in at least one EV condition were kept for further indepth analysis. RNA coverage visualization was performed with the Integrative Viewer Software (IGV; Thorvaldsdóttir et al., 2013) on a log scale.

Subcellular location prediction was performed with SurfG+ (Barinov et al., 2009). Clusters of Orthologous Groups (COGs) and KEGG categories were obtained using the eggNOG-mapper v2 web tool (Huerta-cepas et al., 2017, 2019). Functional enrichment analysis was performed with g:Profiler web-server (Raudvere et al., 2019; Reimand et al., 2019). A maximum value of *p* 0.05 was set as a threshold for significative categories.

A timepoint clustering study was conducted with the R package maSigPro (Conesa et al., 2006; Nueda et al., 2014) on highly covered EV RNAs with normalized counts by EdgeR. In this analysis vancomycin treatment is not taken into consideration. The threshold of statistical significance was set to 0.05, with the adjustment method of Benjamini-Hochberg.

### RT-qPCR

Reverse transcription quantitative PCR (RT-qPCR) was used to validate RNA-seq results. EVs were isolated from the cell-free supernatants of three new independent *S. aureus* cultures at late-stationary growth phases (12 h) in the absence of vancomycin. EV RNAs were purified as mentioned above. Around 1.5 μg of RNAs was treated with DNAse I (Amplification Grade, Invitrogen) according to manufacturer’s instructions. cDNA synthesis was performed with the high capacity cDNA Reverse Transcription kit (Applied Biosystems). The primers used for quantitative PCR (qPCR) are listed in Supplementary Table S2 and were designed using eprimer3 software (EMBOSS). qPCR was carried out in a 16 μl volume containing 15 ng cDNA, specific primers (300 nM), and 8 μl IQ™ SYBR Green Supermix (Bio-Rad). Reactions were run on a CFX96 real-time system (Bio-Rad, France) using the following cycling parameters: DNA polymerase activation and DNA denaturation 95°C for 5 min, 40 cycles of denaturation at 94°C for 15 s, and extension at 60°C for 30 s. Melting curve analysis was included to check the amplification of single PCR products. Samples setups included biological triplicates and technical duplicates as well as negative controls corresponding to qPCR reactions performed without cDNA (cDNA negative control) and from RT reactions obtained from EV RNAs without reverse transcriptase (RT negative control). Results were analyzed with the GFX Manager software and Ct values were determined. Results with Ct equal or above 40 were considered negative and only experiments with ΔCt ≥ 4 between negative controls and RT samples were considered.

## Results

### *S. aureus* HG003 Produces EVs in Different Growth Conditions

**Figure 1:**
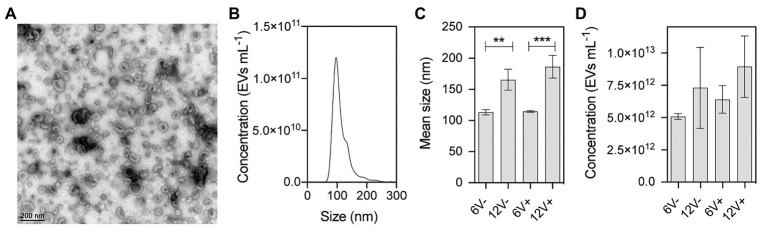
Physical characterization of purified *Staphylococcus aureus* HG003-secreted extracellular vesicles (EVs). **(A)** Representative electron microscopy image of negatively stained HG003 EVs. **(B)** Representative graph of the EV size distribution. **(C)** Mean EV sizes. **(D)** EV yields. Data were obtained from three independent EV replicates. Asterisks indicate statistical significance (one-way ANOVA followed by Tukey’s multiple comparisons test: ^**^p < 0.01; ^***^p < 0.001). Early- and late-stationary growth phases (6 and 12, respectively) in the absence (V−) or presence (V+) of vancomycin.

Extracellular vesicles secreted by HG003 were isolated from the cell-free supernatants of bacterial cultures at early- and late-stationary growth phases (6 and 12 h, respectively), as well as in the absence (V-) and presence (V+) of a sublethal concentration of vancomycin (0.5 μg/ml). For that purpose, we used centrifugation, filtration, and density gradient ultracentrifugation, the standard method for EV isolation and purification at high purity (Yamada et al., 2012; Dauros Singorenko et al., 2017). EV homogeneity and integrity were evaluated by both negative staining electron microscopy and by NTA. Electron micrographs of purified EVs revealed typical nano-sized vesicular structures, with cup-shaped forms in all tested conditions (Raposo and Stoorvogel, 2013; Figure 1A and Supplementary Figure S2). NTA analyses showed a typical profile of particles for all EV samples (Figure 1B and Supplementary Figure S2). A significant increase of approximately 55% in EV diameter was observed in those purified from late-stationary phase cultures, compared to early-stationary phase cultures (for both 6V− vs. 12V− and 6V+ vs. 12V+), whereas no significant difference was observed in the absence or presence of vancomycin (6V− vs. 6V+ and 12V− vs. 12V+, Figure 1C). EV yield is essentially similar at the two growth phases, irrespective to the presence/absence of vancomycin (Figure 1D). In summary, *S. aureus* HG003 releases EVs with variable diameters depending on the growth phase. A sublethal concentration of vancomycin, however, does not impact the EV morphology, concentration, or diameter.

### The *S. aureus* EVs Harbor all RNA Functional Classes

**Figure 2:**
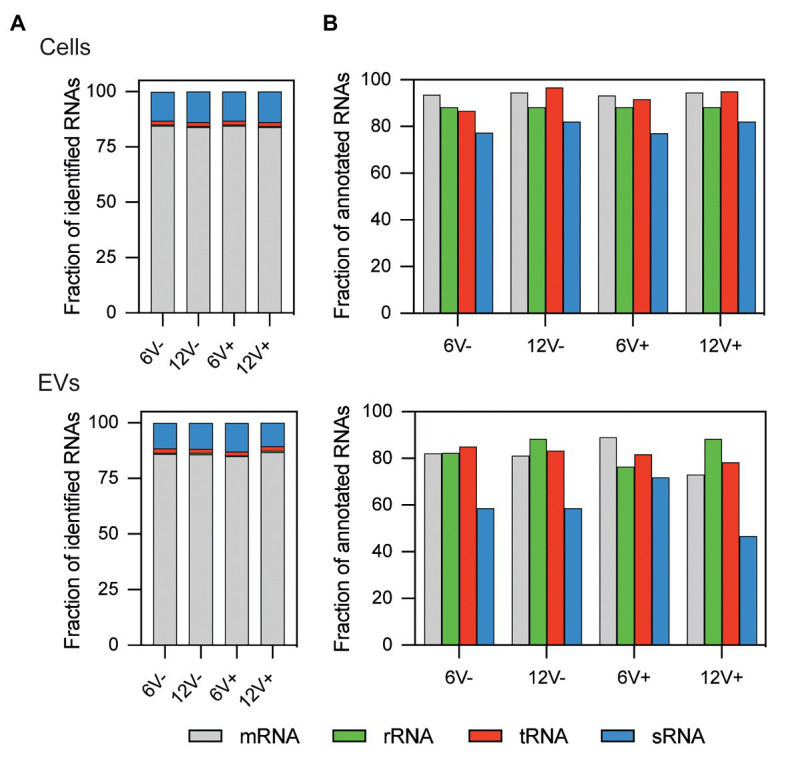
Relative RNA composition of *S. aureus* HG003 and its secreted EVs. Individual colored bars represent the relative amount of each RNA class for mapped reads **(A)** and annotated RNAs **(B)**. RNA-Seq data is the average of three independent replicates. Number of reads have been normalized with EdgeR. RNA classes are defined from the *S. aureus* genome annotation NCTC8325/HG003. Early- and late-stationary growth phases (6 and 12, respectively) in absence (V−) or presence (V+) of vancomycin.

Total RNA was extracted from HG003 EVs to investigate their compositions. The quality of the RNA preparations was checked and validated, and the samples sequenced. RNA-seq data were compared between the different growth conditions, and with those obtained from parental HG003 cells that produced the EVs in each condition (i.e., the EV-producing cells). Around 2649 ± 238 RNAs annotated in the HG003 genome were identified in the EVs according to growth conditions, with an average count of over five reads per RNA in each condition, whereas 3120 ± 35 annotated RNAs were identified within the EV-producing cells (Supplementary Tables S3 and S4). All the four main RNA functional classes (tRNAs, rRNAs, mRNAs, and sRNAs) were identified in both the purified EVs and the EV-producing cells (Figure 2A). In both the EVs and the EV-producing cells, ~84% of the mapped RNAs corresponded to protein-coding genes (mRNAs). The sRNAs were the second most abundant mapped RNA class (~12% of the reads). The remaining 4% of the mapped RNAs are tRNAs and residual rRNAs. Note that most of the rRNAs were voluntarily removed during the RNA purification. Most of the annotated mRNAs, tRNAs, and residual rRNAs were identified in the EV-producing parental cells (from 86 to 96%), while this value dropped to 79.8 ± 2.7% for sRNAs (Figure 2B). These percentages were slightly lower for EV samples (from 72 to 89%), although they remained high for the sRNAs (59.1 ± 10.2%). All these RNAs detected in the purified EVs prompted us to check their coverages, to evaluate their integrity.

### The *S. aureus* Purified EVs Contain Both Fragmented and Intact RNAs From Various Functional Classes

**Figure 3:**
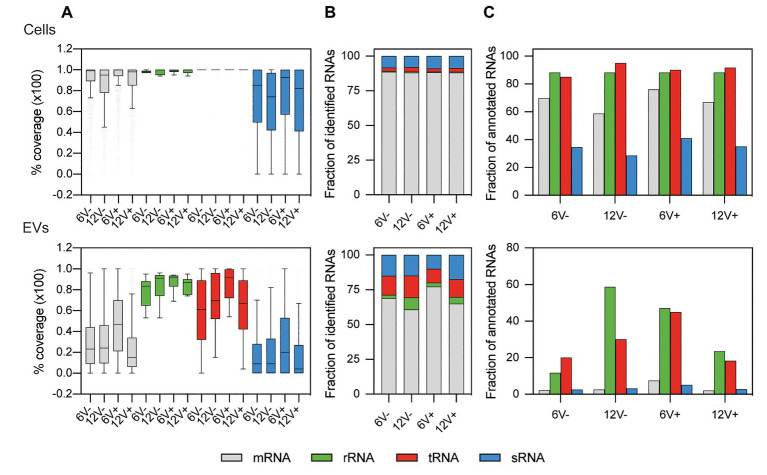
Relative composition of highly covered RNAs from *S. aureus* HG003 and its secreted EVs. Colors represent the relative amount of each RNA class. **(A)** Percentage of RNA median coverage. Distribution of newly filtered RNAs with ≥90% coverage were plotted for **(B)** mapped reads **(C)** and annotated RNA. Number of reads have been normalized with EdgeR. RNA classes are defined from the *S. aureus* genome annotation NCTC 8325/HG003. Early- and late-stationary growth phases (6 and 12, respectively) in absence (V−) or presence (V+) of vancomycin.

For the EV-producing cells, the median values of mRNAs, tRNAs, and residual rRNAs coverages were between 95 and 100%, and were 74 and 92% for the sRNAs (Figure 3A and Supplementary Table S4), implying that those RNAs were mainly intact, and not degraded. The coverage profile was drastically different for the RNAs recovered from the purified EVs. While the coverage of the residual rRNAs varied from 83 to 92%, the median coverage values for the other RNA functional classes ranged from 61 to 92% for the tRNAs, 15 to 47% for the mRNAs, and 4 to 20% for the sRNAs (Figure 3A). These lower coverages suggested that a substantial fraction of mRNAs and sRNAs were fragmented in the EVs compared to the EV-producing cells. To analyze potentially functional RNAs in EVs, only RNAs with a coverage ≥90% were considered for further analysis (Supplementary Table S5). The distribution of the newly filtered RNAs was depicted in Figures 3B,C. Such a harsh quality criterion impacted mainly the RNAs from the EVs, and particularly mRNAs and sRNAs. Only 3.5 ± 2.7 and 3.4 ± 1.2% of annotated mRNAs and sRNAs, respectively, were identified within EVs with such a threshold, while 67.9 ± 7.2% and 34.9 ± 5.0% of annotated mRNAs and sRNA were identified, respectively, for EV-producing cells (Figure 3C). Compared to the parental cells, the EVs were slightly depleted into mRNAs (68.0 ± 7.0% for the EVs vs. 88.2 ± 0.3% in the EV-producing cells) but, interestingly, were enriched for the other RNA functional classes including the sRNAs (14.3 ± 3.2% for the EVs vs. 8.6 ± 0.4% in the EV-producing cells, Figure 3B).

### Functional Characterization of the RNAs From the EVs

**Figure 4:**
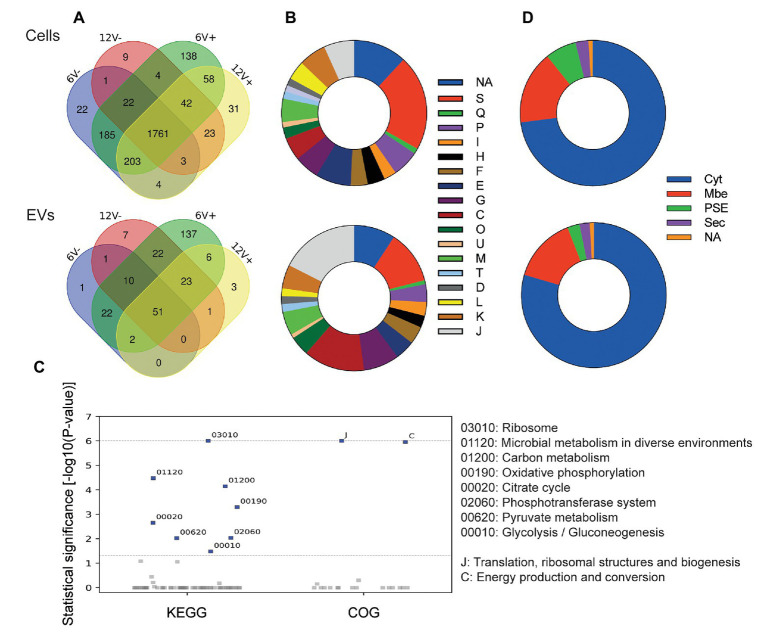
*Staphylococcus aureus* HG003 EV RNA cargo and its modulation by different growth conditions. **(A)** Venn diagrams of RNA composition in EV-producing cells (upper panel) and EVs (lower panel) from different growth conditions. Early- and late-stationary growth phases (6 and 12, respectively) in absence (V-) or presence (V+) of vancomycin. **(B)** Prediction of Clusters of Orthologous Groups (COG) categories for mRNAs: NA, not predicted; S, function unknown; Q, secondary metabolites biosynthesis, transport, and catabolism; P, inorganic ion transport and metabolism; I, lipid transport and metabolism; H, coenzyme transport and metabolism; F, nucleotide transport and metabolism; E, amino acid transport and metabolism; G, carbohydrate transport and metabolism; C, energy production and conversion; O, post-translational modification, protein turnover, and chaperones; U, intracellular trafficking, secretion, and vesicular transport; M, cell wall/membrane/envelope biogenesis; T, signal transduction mechanisms; V, defense mechanisms; D, cell cycle control, cell division, chromosome partitioning; L, replication, recombination and repair; K, transcription; J, translation, ribosomal structure and biogenesis. **(C)** COG and KEGG categories enriched (*p* < 0.05) in EVs compared to EV-producing cells. **(D)** Subcellular localization of proteins encoded by mRNAs as predicted by SurfG+: Cyt, cytoplasmatic; Mbe, membrane; PSE, surface-exposed; Sec, secreted; NA, Not predicted.

**Figure 5:**
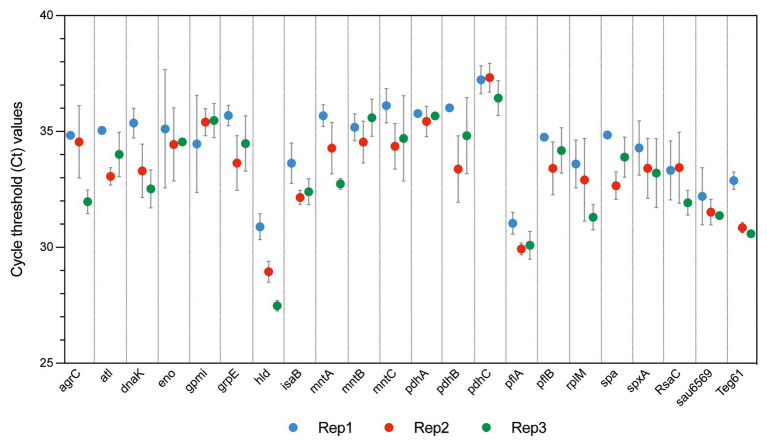
Reverse transcription quantitative PCR (RT-qPCR) validation of *S. aureus* HG003 EV RNAs. RT-qPCR experiments were performed from RNAs extracted from EV samples isolated from the cell-free supernatants of three independent *S. aureus* cultures at late-stationary growth phases (12 h) in the absence of vancomycin (Rep1, Rep2, and Rep3). Quantitative PCR (qPCR) successfully amplified the coding-sequence of 19 mRNAs, and 3 sRNAs. Samples setups included biological triplicates (Rep1, Rep2, and Rep3) and technical duplicates as well as negative controls corresponding to qPCR reactions performed without cDNA, and from RT reactions performed without reverse transcriptase enzyme. Ct values are expressed as mean ± SD from two independent technical replicates performed in triplicates.

**Figure 6:**
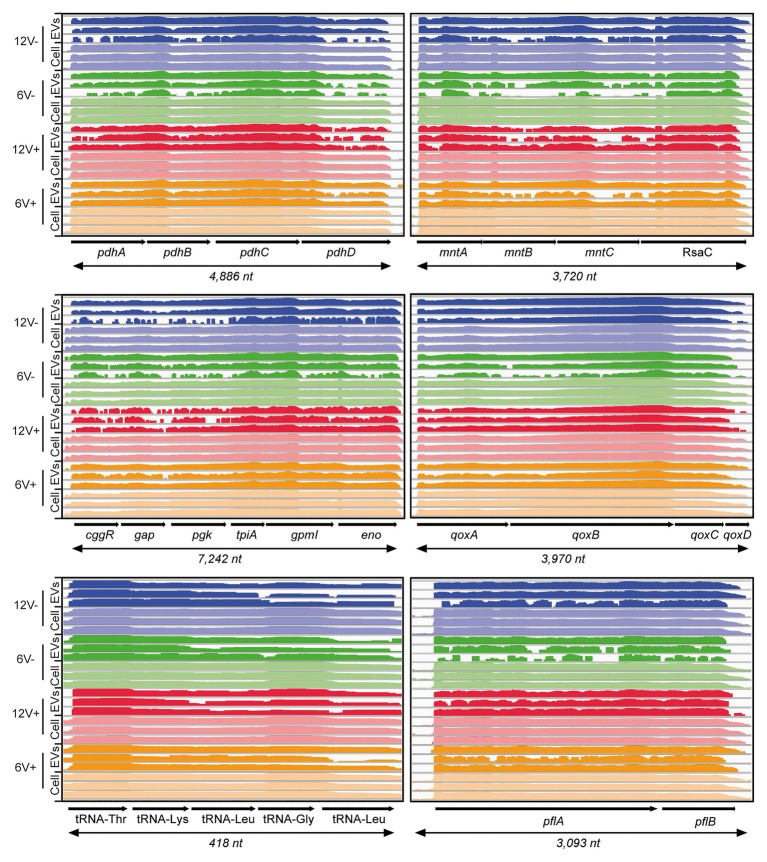
HG003 EVs contain long mRNA transcripts. Comparison between operon coverages between EV-producing cell and EV samples in different conditions. Early- and late-stationary growth phases (6 and 12, respectively) in absence (V-) or presence (V+) of vancomycin. RNA coverage is visualized with Integrative Viewer Software (IGV) in log scale.

According to experimental conditions, from 86 to 273 RNAs with a ≥90% coverage and an average count of over 5 reads per RNA were identified within EVs from *S. aureus* HG003 (Figure 4A and Supplementary Table S5). They corresponded to 286 unique RNAs and were either mRNAs (220), tRNAs (28), residual rRNAs (10), and sRNAs (28). The presence of some of these transcrits associated with HG003 EVs and corresponding either to mRNAs or sRNAs was confirmed by RT-qPCR on RNAs extracted from three independent biological replicates (Figure 5). Among the mapped mRNAs, most were implicated in translation, ribosomal structure and biogenesis (17.5%, COG J), energy production and conversion (13.6%, COG C), carbohydrate transport and metabolism (COG G, 7.9%), transcription (5.2%, COG K), and cell wall/membrane/envelope biogenesis (5.2%, COG M; Figure 4B). Several COG and KEGG categories were notably enriched (*p* < 0.05) in the EVs compared to the EV-producing bacteria (Figure 4C). mRNAs expressing proteins with a cytoplasmic location prediction were more represented in the EVs (79.5%) than into the producing cells (72.9%; Figure 4D). Interestingly, EVs contained several mRNAs coding for virulence-associated proteins such as the immune evasion protein A and Sbi, the Atl autolysin, the Hld δ-hemolysin encoded by the multifunctional sRNA RNAIII, the PSMβ1 Phenol Soluble Modulin, the FntA iron-storage ferritin, and the MntABC iron ABC transporter. Among the 20 tRNAs annotated in the genome, 15 were identified into the EVs (tRNA^His^, tRNA^Asn^, tRNA^Glu^, tRNA^Arg^, and tRNA^Asp^ were absent). Five copies of the 16S and 23S rRNAs were also detected, implying that our rRNA depletion procedure was incomplete. Finally, 28 annotated potential sRNAs were detected within EVs, and among the 50 or so *bona fide* sRNAs defined for the HG003 strain (Liu et al., 2018a), only RsaC was identified with a ≥90% coverage in this study. Note that despite encoding the highly covered Hld transcript, the sRNA RNAIII presented only 71% gene coverage and therefore was excluded from analysis. Around 196 out of the 286 EV-associated RNAs colocalized at the same loci onto the HG003 chromosome, to form 42 clusters of 2 to 29 contiguous genes that were experimentally shown to belong to the same transcriptional units (Mäder et al., 2016; Supplementary Table S6). Among these transcriptional units, 17 displayed a RNA-Seq coverage ≥90% across the entire operon in both the EVs and the EV-producing bacteria. Figure 6 illustrates the sequencing coverage of various contiguous genes within the EVs and the EV-producing cells. Long mRNA operons, up to ~14,000 nucleotides, were detected as fully covered by reads into the purified EVs, supporting the presence of highly covered RNAs and operons as full-length transcripts.

### EV RNA Composition Varies With Growth Conditions

The RNA composition of the purified EVs was compared between early- and late-stationary phases, and with or without vancomycin. Eighteen percentage (*n* = 51) of all detected RNAs with ≥90% coverage were common to all the EV samples (Figure 4A and Supplementary Table S5), implying that the RNA content of the EVs highly varied according to the growth conditions. The percentage of RNAs shared by all the EV-producing cell samples, however, was much higher (70%, *n* = 1761). The shared RNAs among the EVs included mRNAs expressing virulence factors (Atl and Spa), metabolic enzymes (pyruvate dehydrogenase and cytochrome c oxidase complexes, glycolytic enzymes) and transcriptional regulators (SpxA, CggR, and GlnR), as well as RNAs involved in translation (ribosomal proteins, rRNAs, and tRNAs; Supplementary Table S5). The 51 common RNAs also included 9 potential sRNAs, notably RsaC involved in *S. aureus* oxidative stress adaptation and nutritional immunity (Lalaouna et al., 2019).

For both the EVs and EV-producing cells, more RNAs were detected at 6 h (275 and 2443 for EVs and EV-producing cells, respectively) than at 12 h (126 and 2161 for EVs and EV-producing cells, respectively, Figure 4A). Likewise, more RNAs were detected in the EVs in the presence (277 and 2,474 for EVs and EV-producing cells, respectively) than in the absence of vancomycin (140 and 2,279 for EVs and EV-producing cells, respectively; Figure 4A), indicating that the antibiotic modifies the RNA cargo of the EVs. These results also highlighted that the growth phase and the antibiotic stress impacted mostly the RNA content of the EVs, but much less that of the parental cells. Indeed, when we considered the RNAs detected in only one condition (i.e., specific RNAs), their fractions were higher in the EVs than in EV-producing cells, and that for all the tested conditions. For example, 58% of RNAs found within EVs at 6 h were specific to this condition, while specific RNAs represented only 14% of all RNAs detected at 6 h in the EV-producing cells.

### EV RNA Abundance Varies With Growth Conditions

**Figure 7:**
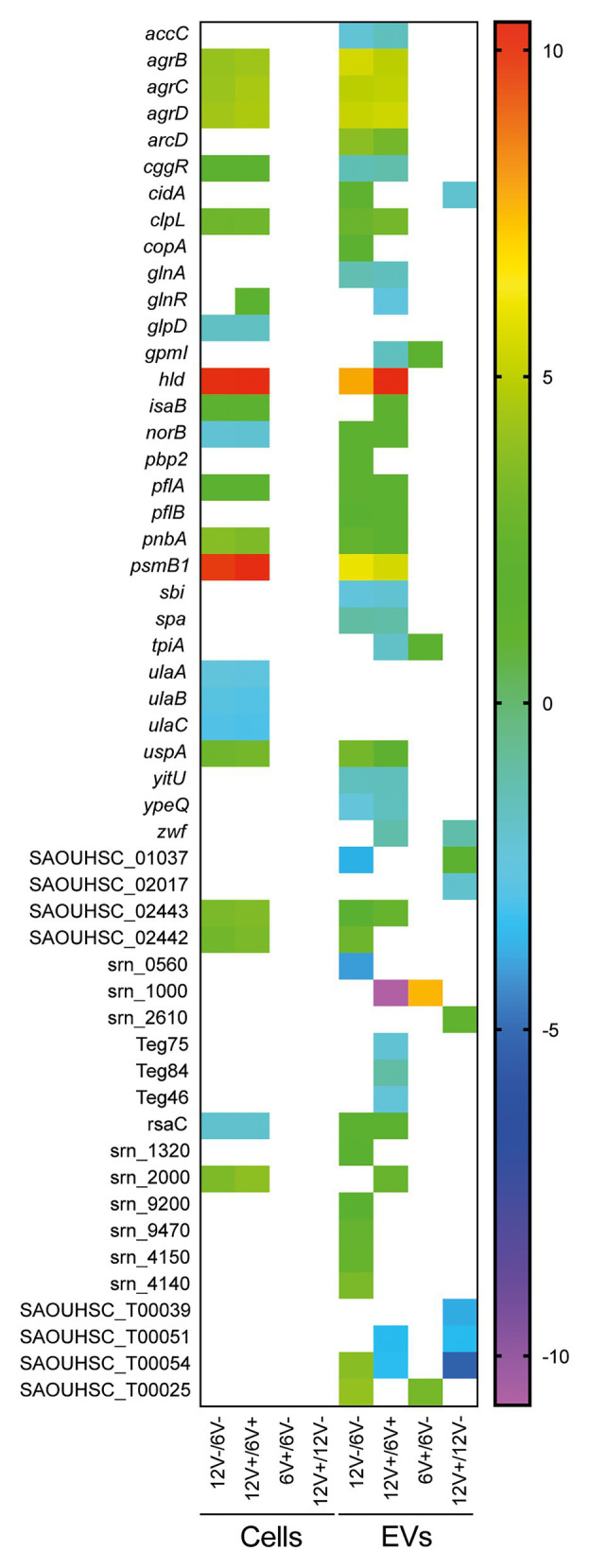
Differential RNA abundance between the EV-producing cells and the EVs. Colored square shows different RNA abundance patterns between EV-producing cells and EVs with a log2 fold change. Comparisons comprised data with at least one of the two samples containing ≥90% coverage. The log2 fold change is displayed as colored squares from −2 (purple) to 10 (red). Early- and late-stationary growth phases (6 and 12, respectively) in absence (V−) or presence (V+) of vancomycin.

In addition to the qualitative variations observed, significant differences (Padj < 0.05) in EV RNA abundance between the experimental conditions were also detected (Supplementary Table S7). Among the 286 EV-associated RNAs, 110 were differentially abundant between two conditions. Variations were detected at all times and in the absence or presence of vancomycin, although the growth phase appeared to have a greater impact on RNA abundance (75 and 64 differentially abundant RNAs were detected between early- and late-stationary phase with or without vancomycin, respectively), than the antibiotic stress (8 and 9 differentially abundant RNAs were detected between presence and absence of vancomycin in early- and late-stationary phase, respectively; Supplementary Table S7). A selection of RNAs with a modulation of their abundance according to the growth conditions is displayed in Figure 7. The most modulated RNAs into EVs produced from the two growth conditions were mRNAs coding for virulence-associated factors, such as *agrB*, *agrC*, *agrD*, *psmB1*, and *hld* with a 30- to 1300-fold change, two potential annotated sRNAs, srn_0560, and srn_1000 with a 16- and 190-fold change, respectively, tRNA^Gly^ (SAOUHSC_T00025) and tRNA^Thr^ (SAOUHSC_T00054), with fold changes greater than 16. Among the differentially abundant RNAs according to the growth phase, 32 were detected both in presence and absence of vancomycin, with similar fold changes highlighting their reproducible variations into EVs across different environmental conditions (Figure 7 and Supplementary Table S7).

Differentially expressed RNAs were also detected for the EV-producing cells when their expression was compared between early- and late-stationary phase both in absence (*n* = 136) and in presence of vancomycin (*n* = 147), which was expected since bacterial transcription differs qualitatively and quantitatively when facing different growth conditions (Supplementary Table S8). Note that no significantly differentially expressed RNAs were detected according to the presence of vancomycin. As observed previously for the EV RNA content, the growth conditions, particularly the growth phase, impacted mostly the RNA abundance of the EVs, but much less than of the parental cells. Indeed, 38% of RNAs detected within EVs displayed changes in their abundance between conditions, while the fraction of modulated RNAs counted for only 2% of the RNAs in the EV-producing cells. The abundance pattern of several RNAs differed between the EVs and the EV-producing cells according to the growth conditions (Figure 7). While some RNAs such as *agrBCD*, *psmβ1*, and *hld* mRNAs displayed the same variations of their abundance pattern in EVs and EV-producing cells regardless the growth conditions. Others, such as *spa* and RsaC, were differentially abundant between the EVs and the EV-producing cells.

### Timepoint Clustering Analysis Reveals Different RNA Abundance Profiles Between the EVs and EV Producing Cells

**Figure 8:**
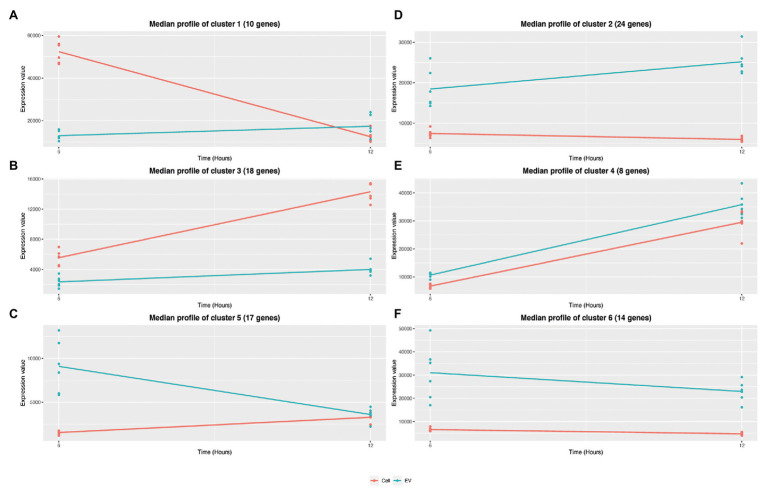
Timepoint clustering of expression profiles between EVs and EV-producing cells. The analyses were performed with the R package maSigPro from normalized counts by EdgeR. Statistical significance (*p* < 0.05) was adjusted with Benjamini-Hochberg method. Median expression values are calculated at 6 and 12 h for EVs (blue) and EV-producing cells (red) for the six clusters (from A to F).

To evaluate the influence of the growth phase on EV and EV producing cell RNA composition, a negative binomial-based approach, with the R package maSigPro (Conesa et al., 2006) was applied. Briefly, maSigPro provides a differentially expressed transcript analysis of serial data between experimental groups (e.g., EV and EV producing cells). maSigPro was applied to the 286 highly covered EV RNAs and identified 91 RNAs with significant temporal profile changes (Padj < 0.05). RNAs were clustered according to their expression profiles (Supplementary Table S9). Figure 8 shows the six RNA clusters obtained. Three clusters grouped transcripts with a similar expression profile between EV and EV-producing cells: cluster 4 with 8 RNAs (including, e.g., *agrBD* and *arcC2*) and cluster 3 with 18 RNAs (including, e.g., *hld* and *psmβ1*) contained more abundant transcripts over time in both EVs and EV-producing cells, whereas cluster 6 with 14 RNAs (including, e.g., *fusA*, *tuf*, and *secY1*) contained less abundant transcripts at 12 h than at 6 h in both. Interestingly, the three other clusters grouped RNAs that showed opposite expression profiles over time in EVs and EV-producing cells. Cluster 1 with 10 RNAs (including, e.g., RsaC and *pdhA*), and cluster 2 with 24 RNAs (including, e.g., *ldh1*, *qoxABC*, and *rpoBC*) grouped, similarly, more abundant transcripts at 12 h in EVs, and less abundant transcripts at 12 h in EV-producing cells. On the contrary, cluster 5 with 17 RNAs (including, e.g., *ccgR* and *sbi*) grouped less abundant transcripts in EVs and more abundant transcripts in EV-producing cells at 12 h. Altogether, this analysis highlighted that the transcript expression pattern could temporally differ between EVs and EV-producing cells.

## Discussion

Extracellular vesicles are universal carriers of macromolecules including extracellular RNAs all along from bacteria, archaea, and fungi to protists. Recent investigations on bacterial EV biogenesis, release, and trafficking showed their functional importance for bacterial communication and survival (Tsatsaronis et al., 2018). Information regarding *S. aureus* EV RNA cargo, however, is lagging behind. Here, we report the first exploratory work on EVs released by *S. aureus* HG003, the characterization of its EV RNA cargo under different conditions, and an indepth transcriptomic comparison between the EVs and the EV-producing cells.

Environmental conditions, such as growth phase and environmental stresses, reportedly influence the production, content and functions of EVs (Tashiro et al., 2010; Kim et al., 2016b; Orench-Rivera and Kuehn, 2016; Askarian et al., 2018; Yun et al., 2018; Andreoni et al., 2019). Here, to investigate the impact of environmental changes on *S. aureus* EV production, the selected conditions were the early- and late-stationary growth phases, with or without a sublethal concentration of vancomycin that does not impact growth. Differences were observed regarding EV sizes. EVs derived from the late-stationary growth phase were larger than those collected during the early-stationary phase. This can be due to cell-wall morphology and peptidoglycan structure that are characteristics of the growth stage in *S. aureus* (Zhou and Cegelski, 2012). A correlation between the degree of peptidoglycan cross-linking to the cell wall stiffness and EVs release was observed for both Gram-negative and Gram-positive bacteria (Zhou et al., 1998; Deatherage et al., 2009; Schrempf and Merling, 2015; Schwechheimer et al., 2015; Wang et al., 2018). Notably, sub-inhibitory concentrations of penicillin decreases peptidoglycan cross-linking, triggering an increase in *S. aureus* EV yields and sizes (Wang et al., 2018). Here, we show that sublethal concentrations of vancomycin, an antibiotic that also targets peptidoglycan synthesis in *S. aureus*, does not impact either EV morphology or EV production yields, but does change their RNA content in terms of composition and abundance. Vancomycin affects *S. aureus* EV activity starting at 1 μg/ml (He et al., 2017). The sub-inhibitory vancomycin concentration used here (0.5 μg/ml) is probably too low to detect any changes in EV morphology and production.

All RNA classes are detected by RNA-Seq within HG003-derived EVs. These include rRNAs, which were still detected, suggesting that the rRNA depletion carried out here was incomplete. In the absence of filtering by coverage of sequencing data, on average, 78.0 ± 7.0% of the annotated transcripts in HG003 genome were present in the EVs (91.8 ± 1.0% for EV-producing cells). Of these, a large portion of mapped RNAs corresponded to mRNAs. These results are consistent with RNA-Seq data obtained with similar criteria from OMVs in *Salmonella enterica* serovar Typhimurium (*S. Typhimurium*) that harbor around 73% of the annotated transcripts including up to 86% of mRNAs according to growth conditions (Malabirade et al., 2018). A recent study addressing the sRNA content of EVs also pointed out that mRNAs are the more abundant RNA species in EVs derived from *S. aureus* strain MSSA476 after rRNA depletion (Joshi et al., 2021). On the contrary, studies with *Escherichia coli* revealed that EVs were enriched mainly with short RNAs, such as tRNAs (Ghosal et al., 2015; Blenkiron et al., 2016). Nevertheless, these variations may be a result of different RNA extraction and library preparation protocols, or simply correspond to singular characteristics of EVs derived from different bacterial species. As expected, data filtering of RNAs with ≥90% coverage decreased the number of detected RNAs. However, the RNA content of the EVs, particularly the mRNAs and sRNAs (only 5.1 ± 2.8% of EV RNAs initially detected are still identified after the filtering), was much more affected than that of the EV-producing cells (69.0 ± 7.90% of EV-producing cell RNAs are still detected after the filtering). The low RNA coverage in the HG003 EVs might perhaps reflect the absence of transcription within EVs and, thus, the progressive degradation of a substantial fraction of the EVs-associated RNAs after their formation and/or during their purification. These findings are consistent with a recent report showing that the predominant RNA type in EVs from *S. aureus* Newman is <300-nucleotide long (Rodriguez and Kuehn, 2020). The presence of numerous processed or degraded RNAs could be a common feature of the bacterial EVs, as Gram-negative *S. Typhimurium* OMV-associated RNAs are also processed or degraded (Malabirade et al., 2018). Since one of the primary physiological functions attributed to EVs is the removal of unwanted materials from cells, such as misfolded or degraded proteins (McBroom and Kuehn, 2007), the *S. aureus* EVs may also help removing the degraded RNAs from the bacteria (Groot and Lee, 2020).

Two hundred and eighty-six highly covered RNAs can be identified within HG003 EVs. That number of highly covered EV RNAs varies according to growth conditions, from 86 in late-stationay growth phase with vancomycin to 273 in early-stationay growth phase also with vancomycin. As expected, all the EV transcrits are also detected in the EV-producing cells. Among them, 51 transcripts are shared by EVs collected in all tested conditions. The 286 highly covered EV RNAs encompass short transcripts, such as tRNAs (~75 nucleotides), mRNAs (*psmβ1*, 135 nucleotides; *agrD*, 141 nucleotides), and some sRNAs (Teg84, 79 nucleotides; Teg46, 124 nucleotides). Yet, these highly covered EV RNAs also comprise long transcripts, including 15 mRNAs with lengths >2,000 nucleotides (e.g., *pbp2*, *copA*, *rpoB*, *rpoC*, and *atl*). 67% of these RNAs are organized into 42 gene clusters that are co-transcribed in *S. aureus* (Mäder et al., 2016). Among them, 17 are full-length transcripts across entire operons in HG003 EVs, with lengths up to ~14,000 nucleotides. As observed for *S. Typhimurium* OMV RNAs (Malabirade et al., 2018), these findings support that highly covered RNAs are present as full-length transcripts. These RNAs belong to all annotated classes of RNAs. The mRNAs from the EVs encode proteins involved in transcription, translation, energy production and conversion, carbohydrate metabolism, and cell wall biogenesis. In addition to these housekeeping functions, EVs also harbors mRNAs encoding virulence-associated proteins, such as the *agr* operon responsible of quorum-sensing, autolysin Atl, protein A (Spa), immunoglobulin-binding protein (Sbi), immunodominant staphylococcal antigen B (IsaB), δ hemolysin (Hld) encoded by the multifunctional sRNA RNAIII, and the PSMβ1 phenol-soluble modulin, as well as several iron acquisition systems. Besides, most tRNA species are detected within the EVs, as well as residual rRNAs and 28 annotated sRNAs, including *bona-fide* RsaC involved in *S. aureus* oxidative stress adaptation and nutritional immunity (Lalaouna et al., 2019). Note that RsaC and RNAIII were also detected within *S. aureus* EVs from strain MSSA476 (Joshi et al., 2021), suggesting their wider occurence in staphylococcal EVs.

The presence of full-length, functional RNAs in EVs raises the question of their biological roles. EVs are produced to transport bioactive molecules to interact and communicate with other cells. So far, most studies on *S. aureus* EVs investigated the protein cargo. Therefore, the broad spectrum of activities associated with *S. aureus*-derived EVs was related to their protein content (Jeon et al., 2016). In some *S. aureus* strains, EVs carry β-lactams that confer transient resistance to ampicillin-susceptible *E. coli* and *S. aureus* (Lee et al., 2013b). Likewise, mycobactin-containing *Mycobacterium tuberculosis* EVs can deliver iron to strains deficient for iron-uptake (Prados-Rosales et al., 2014). The delivery of full-length mRNAs *via* EVs to the surrounding bacterial cells, notably those involved into energetic and metabolic functions, could improve their responses to environmental stimuli to fasten their adaptation. Likewise, rRNAs and tRNAs could boost translation in EV recipient bacterial cells and improve their fitness. In bacteria, sRNAs fine tune target gene expression, usually at the posttranscriptional level in response to changes in the environment, including antibiotic resistance and tolerance (Mediati et al., 2021). Most bacteria encode dozens of sRNAs that are transcribed as independent transcripts or processed from mRNAs. The presence of sRNAs with regulatory roles within *S. aureus* EVs could be a relocation strategy in other surrounding bacteria that need more of these sRNAs for adaptation and infection spreading and/or to coordinate bacterial group adaptation, activities and behaviors. The transfer of RsaC could enhance the concentration of that riboregulator to other *S. aureus* cells when intracellular, especially helping survival within the phagolysosome if the *S. aureus* EVs are internalized by the host cells together with the bacteria. Transfer of functional RNAs to bacterial cells that do not encode the corresponding genes in their genome could also be part of a transient horizontal phenotype acquisition, which could be of use during infection to disseminate specific virulence-associated factors through the bacterial community. Finally, beside interactions between bacterial cells, *S. aureus* EV associated RNAs, notably sRNAs, may be involved in the host-pathogen interactions (Eberle et al., 2009; Li and Chen, 2012; Furuse et al., 2014; Sha et al., 2014; Koeppen et al., 2016; Westermann et al., 2016; Choi et al., 2017; Frantz et al., 2019; Han et al., 2019; Lee, 2019; Rodriguez and Kuehn, 2020). The 28 potential annotated sRNA detected within HG003 EVs are potential candidates for further functional characterization, especially during *S. aureus*-host cell interactions. *S. aureus* secreted EVs elicit immune responses that mimic those of the EV-producing cells (Gurung et al., 2011; Hong et al., 2011, 2014; Kim et al., 2012, 2019; Thay et al., 2013; Choi et al., 2015; Jeon et al., 2016; Jun et al., 2017; Askarian et al., 2018; Tartaglia et al., 2018; Wang et al., 2018, 2020; Rodriguez and Kuehn, 2020). Strikingly, within their RNA cargo, several mRNAs encode immunomodulatory proteins, as Sbi, Spa and PSMβ1, and may participate into the immune response triggered by the protein cargo if they are ultimately translated. Yet, such functions remain to be demonstrated for the RNA cargo of *S. aureus* EVs. mRNAs expressing PSMβ and hemolysin δ toxins from the EVs, if translated into recipient bacteria or host cells, could perhaps facilitate staphylococcal intracellular survival, but this hypothesis should be experimentally challenged.

The RNA cargo of HG003 EVs, in both identity and abundance, depends on the growth conditions. Similarly, the EV RNA cargo of *S. Typhimurium* is also sensitive to environmental changes indicating that it reflects the bacterial adaptation to its environment (Malabirade et al., 2018). It could be a faster way to transfer information of changes perceived by one cell to surrounding cells even before they sensed the environmental stimuli in order to quickly promote group adaptation. We found, however, that the vancomycin treatment had less impact on RNA abundance compared to the growth phase. Although the composition of HG003 EVs represented the intracellular state of the bacterial transcriptome through global packaging, two main findings, however, reinforce the concept of a potential selective packaging of RNAs into EVs, as proposed for its protein cargo (Haurat et al., 2011; Cahill et al., 2015; Tartaglia et al., 2020). First, we measured an enrichment for several functional and subcellular localization RNA categories in EVs when compared to EV-producing cells. Second, the relative abundance of several RNAs between two environmental conditions was different in the EVs and the EV-producing cells. Other studies also found that some RNA populations were enriched in EVs from Gram-negative and Gram-positive pathogenic bacteria (Ghosal et al., 2015; Koeppen et al., 2016; Resch et al., 2016; Malabirade et al., 2018; Malge et al., 2018; Han et al., 2019; Langlete et al., 2019; Zhang et al., 2020). This notably includes sRNAs, which can play regulatory activity in the host (Koeppen et al., 2016; Malabirade et al., 2018; Langlete et al., 2019; Zhang et al., 2020). The enrichment of RNAs associated with bacterial diseases in EVs derived from many pathogenic bacteria reinforces the physiopathological role of these structures in host-pathogen interaction and host cell invasion, which could be borne by their RNA cargo as well as by their protein cargo. The selective mechanisms of EV RNA content packaging have not yet been elucidated. It has been proposed that RNA packing into EVs could depend on RNA size and location (eg., nearby EVs formation site), as well as on their affinity for other molecules (eg., membrane proteins; Langlete et al., 2019). Nevertheless, such enrichment results should be interpreted with carefulness. Indeed, they could also reflect a difference in RNA half-lives between EVs and EV-producing cells, as well as a difference in RNase activity (Langlete et al., 2019; Lécrivain and Beckmann, 2020), pointing out that RNAs with longer half-lives could be protected from degradation, leading to an artifactual accumulation in the EVs over time.

In summary, our exploratory work provides novel insights in *S. aureus* EVs by the characterization of its RNA cargo and paves the way for further functional studies. Mainly, it sheds light on the possible roles of EV RNA cargo in intra- and inter-species communication, in the virulence and pathogenesis of *S. aureus*, and as trash bags for degraded RNAs. The study of bacterial EV RNA cargo is an emerging area of research. Evidently, as with all emerging fields, each advance raises further questions: Are the full-length RNAs in HG003 EVs functional, and do they possess similar functions than in the bacterial cytoplasm? What are the rules for RNA sorting into HG003 EVs? What are the roles and functions of the *S. aureus* EV RNA cargo? These exciting questions, among others, should be adressed in further studies.

## Data Availability Statement

The datasets presented in this study can be found in online repositories. The names of the repository/repositories and accession number(s) can be found at: https://www.ebi.ac.uk/ena, https://www.ebi.ac.uk/ena/browser/view/PRJEB40502.

## Author Contributions

BSRL, BF, SC, YL, VA, and EG conceived and designed the experiments. VA and EG supervised the study. BSRL and SC performed the experiments. AN performed computational analysis. BSRL, BF, SC, VRR, AN, and EG analyzed the data. VA, YL, and EG contributed to funding acquisition. BSRL and EG wrote the original draft. All authors contributed to data interpretation, drafting the manuscript, critically revising the manuscript, and approving its final version.

### Conflict of Interest

The authors declare that the research was conducted in the absence of any commercial or financial relationships that could be construed as a potential conflict of interest.
