# Supplementary material for: Environmental Plasticity of the RNA Content of Staphylococcus aureus Extracellular Vesicles
Source: Front Microbiol. 2021 Mar 11;12:634226. doi: 10.3389/fmicb.2021.634226 (PMC7990786; doi:10.3389/fmicb.2021.634226)
Supplement: Supplementary file 2 [file Presentation_1.pdf]

Supplementary Material

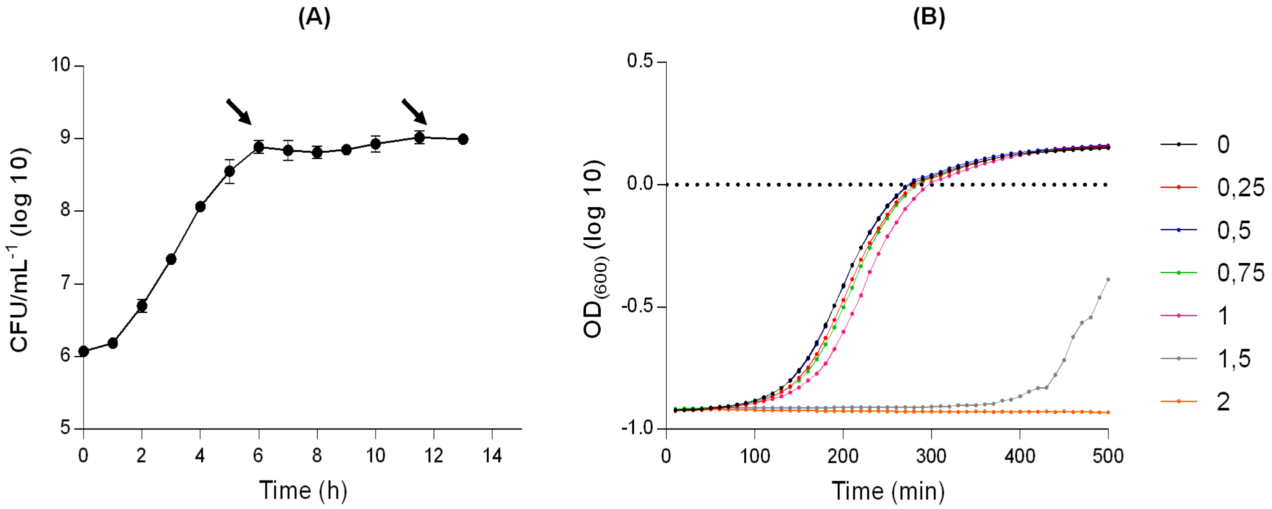

Supplementary Figure 1. Growth curve of *S. aureus* HG003 in absence or presence of increasing concentrations of vancomycin. (**A**) Colony-forming units (CFU) were assessed every hour in cultures in the absence of vancomycin. Black arrows indicate growth phases chosen for RNA characterization (6 h and 12 h). (**B**) Optical density was measured every 10 minutes in different vancomycin concentrations. Black: 0 µg / ml; Red; 0.25 µg / ml; Blue: 0.5 µg / ml; Green: 0.75 µg / ml; Pink: 1 µg / ml; Gray: 1.5 µg / ml; and Orange: 2 µg / ml.

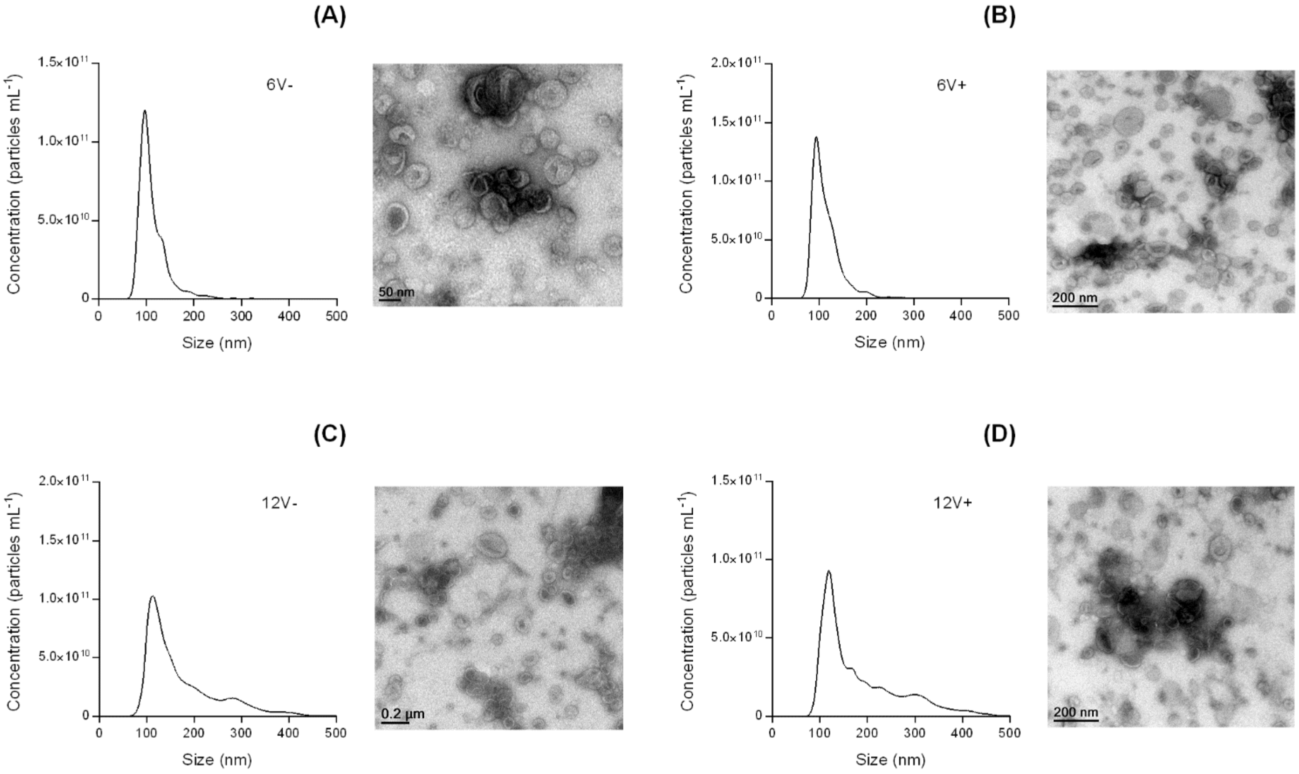

Supplementary Figure 2. NTA profile and electron microscopy of *S. aureus* HG003 derived EVs in various conditions. (**A**) 6V-; (**B**) 6V+; (**C**) 12V-; (**D**) 12V+. Early- and late-stationary phases (6 and 12, respectively) in absence (V-) or presence (V+) of vancomycin
